# Supplementary material for: Developmental programming modulates olfactory behavior in C. elegans via endogenous RNAi pathways
Source: eLife. 2016 Jun 28;5:e11642. doi: 10.7554/eLife.11642 (PMC4924998; doi:10.7554/eLife.11642)
Supplement: Supplementary file 1. — DOI: http://dx.doi.org/10.7554/eLife.11642.042 [file elife-11642-supp1.docx]

**Supplemental File 1**

**Strains used in this study**

N2 Bristol *C. elegans* wild-type isolate

CB1370 *daf-2*(*e1370*) *III*

CB1372 *daf-7*(*e1372*) *III*

CB1386 *daf-5*(*e1386*) *II*

CX10 *osm-9*(*ky10*) *IV*

DR20 *daf-12*(*m20*) *X*

DR27 *daf-16*(*m27*) *I*

EL476 *rrf-2*(*ok210*) *I*

FX01155 *alg-3*(*tm1155*) *IV*

GR1168 *age-1(mg44)/mnC1 dpy-10(e128) unc-52(e444)* *II*

GR1307 *daf-16(mgDf50) I*

GR1311 *daf-3(mgDf90) X*

GR1373 *eri-1*(*mg366*) *IV*

GR1747 *mut-15*(*tm1358*) *V*

GR1823 *mut-16*(*mg461*) *I*

JT709 *pdk-1(sa680)* *X*

MT10430 *lin-35*(*n745*) *I*

NL1810 *mut-16*(*pk710*) *I*

NL1820 *mut-7(pk720)* *III*

NL1838 *mut-14(pk738) V*

NL2098 *rrf-1(pk1417)* *I*

NL2099 *rrf-3(pk1426)* *II*

OH4470 *ttx-3(mg158) X*; *otIs24* [*sre-1p::gfp*]

PFR40 *hpl-2(tm1489)* *III*

PFR60 *hpl-1(tm1624)* *X*

RB774 *zfp-1(ok554)* *III*

RB1024 *drh-2(ok951)* *IV*

RB1025 *set-2(ok952) III*

RB1079 *alg-4(ok1041)* *III*

RB2519 *drh-1(ok3495)* *IV*

SH20 *pdrEx1* [*osm-9p::gfp; unc-122p::dsRed*]

SH21 *pdrEx2* [*osm-9p::gfp; unc-122p::dsRed*]

SH51 *pdrIs1* [*osm-9*p::*gfp; unc-122p::dsRed*] *III*

SH53 *ergo-1*(*tm1860*) *V*; *pdrIs1* [*osm-9*p::*gfp; unc-122p::dsRed*] *III*

SH54 *alg-2*(*ok304*) *II*; *pdrIs1* [*osm-9*p::*gfp; unc-122p::dsRed*] *III*

SH55 *rrf-3(pk1426) II*; *pdrIs1* [*osm-9*p::*gfp; unc-122p::dsRed*] *III*

SH56 *hpl-1*(*tm1624*) *X*; *pdrIs1* [*osm-9*p::*gfp; unc-122p::dsRed*] *III*

SH57 *mut-15*(*tm1358*) *V*; *pdrIs1* [*osm-9*p::*gfp; unc-122p::dsRed*] *III*

SH58 *drh-1*(*ok3495*) *IV*; *pdrIs1* [*osm-9*p::*gfp; unc-122p::dsRed*] *III*

SH59 *hda-2*(*ok1479*) *II*; *pdrIs1* [*osm-9*p::*gfp; unc-122p::dsRed*] *III*

SH61 *drh-2*(*ok951*) *IV*; *pdrIs1* [*osm-9*p::*gfp; unc-122p::dsRed*] *III*

SH63 *daf-3*(*mgDf90*) *X*; *pdrIs1* [*osm-9*p::*gfp; unc-122p::dsRed*] *III*

SH64 *mut-2*(*ne298*) *I*; *pdrIs1* [*osm-9*p::*gfp; unc-122p::dsRed*] *III*

SH67 *rrf-2*(*ok210*) *I*; *pdrIs1* [*osm-9*p::*gfp; unc-122p::dsRed*] *III*

SH68 *daf-12*(*m20*) *X*; *pdrIs1* [*osm-9*p::*gfp; unc-122p::dsRed*] *III*

SH69 *mut-8* / *rde-2*(*ne221*) *I*; *pdrIs1* [*osm-9*p::*gfp; unc-122p::dsRed*] *III*

SH70 *mut-14*(*pk738*) *V*; *pdrIs1* [*osm-9*p::*gfp; unc-122p::dsRed*] *III*

SH71 *rrf-1*(*pk1417*) *I*; *pdrIs1* [*osm-9*p::*gfp; unc-122p::dsRed*] *III*

SH73 *rde-1*(*ne219*) *V*; *pdrIs1* [*osm-9*p::*gfp; unc-122p::dsRed*] *III*

SH74 *mut-16*(*mg461*) *I*; *pdrIs1* [*osm-9*p::*gfp; unc-122p::dsRed*] *III*

SH75 *daf-5*(*e1386*) *II*; *pdrIs1* [*osm-9*p::*gfp; unc-122p::dsRed*] *III*

SH76 *csr-1*(*tm892*) *IV*; *neIs20*; *pdrIs1* [*osm-9*p::*gfp; unc-122p::dsRed*] *III*

SH83 *drh-3*(*ne4253*) *I*; *pdrIs1* [*osm-9*p::*gfp; unc-122p::dsRed*] *III*

SH84 *lin-35*(*n745*) *I*; *pdrIs1* [*osm-9*p::*gfp; unc-122p::dsRed*] *III*

SH85 *daf-16*(*m27*) *I*; *pdrIs1* [*osm-9*p::*gfp; unc-122p::dsRed*] *III*

SH86 *mut-16*(*pk710*) *I*; *pdrIs1* [*osm-9*p::*gfp; unc-122p::dsRed*] *III*

SH87 *nrde-3*(*gg66*) *X*; *pdrIs1* [*osm-9*p::*gfp; unc-122p::dsRed*] *III*

SH88 *alg-4*(*ok1041*) *III*; *pdrIs1* [*osm-9*p::*gfp; unc-122p::dsRed*] *III*

SH89 *daf-7*(*e1372*) *III*; *pdrIs1* [*osm-9*p::*gfp; unc-122p::dsRed*] *III*

SH90 *daf-2*(*e1370*) *III*; *pdrIs1* [*osm-9*p::*gfp; unc-122p::dsRed*] *III*

SH100 *nrde-1*(*gg88*) *III*; *pdrIs1* [*osm-9*p::*gfp; unc-122p::dsRed*] *III*

SH102 *rde-4*(*ne301*) *III*; *pdrIs1* [*osm-9*p::*gfp; unc-122p::dsRed*] *III*

SH103 *nrde-4*(*gg129*) *IV*; *pdrIs1* [*osm-9*p::*gfp; unc-122p::dsRed*] *III*

SH110 *set-2*(*ok952*) *III*; *pdrIs1* [*osm-9*p::*gfp; unc-122p::dsRed*] *III*

SH136 *daf-16*(*mgDf50*) *I*; *pdrIs1* [*osm-9*p::*gfp; unc-122p::dsRed*] *III*

SH137 *eri-1*(*mg366*) *IV*; *pdrIs1* [*osm-9*p::*gfp; unc-122p::dsRed*] *III*

SH138 *eri-6*(*gk3038*) *I*; *pdrIs1* [*osm-9*p::*gfp; unc-122p::dsRed*] *III*

SH157 *zfp-1*(*ok554*) III; *pdrEx1* [*osm-9p::gfp; unc-122p::dsRed*]

SH158 *zfp-1*(*ok554*) III; *pdrEx2* [*osm-9p::gfp; unc-122p::dsRed*]

SH162 *mut-7*(*pk720*) *III*; *pdrEx1* [*osm-9p::gfp; unc-122p::dsRed*]

SH175 *age-1*(*mg44*)/*mnC1* *dyp-10*(*e128*) *unc-52*(*e444*) *II*; *pdrIs1*

[*osm-9*p::*gfp; unc-122p::dsRed*] *III*

SH176 *mut-16*(*mg461*) *I*; *pdrEx15* [*sre-1p::mut-16 cDNA::gfp*;

*unc-122p::dsRed*]

SH177 *mut-16*(*mg461*) *I*; *pdrEx16* [*gpa-4p::mut-16 cDNA::gfp*;

*unc-122p::dsRed*]

SH183 *pdk-1*(*sa680*) *X*; *pdrIs1* [*osm-9*p::*gfp; unc-122p::dsRed*] *III*

SH185 *mut-16*(*mg461*) *I*; *pdrEx18* [*sre-1p::mut-16 cDNA::gfp*;

*unc-122p::dsRed*]

SH186 *mut-16*(*mg461*) *I*; *pdrEx19* [*gpa-4p::mut-16 cDNA::gfp*;

*unc-122p::dsRed*]

SH197 *zfp-1*(*ok554*) *III*; *pdrEx21* [*sre-1p::zfp-1 cDNA::gfp*;

*unc-122p::dsRed*]

SH198 *zfp-1*(*ok554*) *III*; *pdrEx22* [*sre-1p::zfp-1 cDNA::gfp*;

*unc-122p::dsRed*]

SH209 *pdrEx18* [*sre-1p::mut-16 cDNA::gfp*; *unc-122p::dsRed*]

SH211 *pdrEx21* [*sre-1p::zfp-1 cDNA::gfp*; *unc-122p::dsRed*]

SH212 *pdrEx22* [*sre-1p::zfp-1 cDNA::gfp*; *unc-122p::dsRed*]

SH216 *pdrEx19* [*gpa-4p::mut-16 cDNA::gfp*; *unc-122p::dsRed*]

SH225 *daf-3*(*mgDf90*) *X*; *pdrEx41* [*osm-9(mutation 2)::gfp*;

*unc-122p::dsRed*]

SH226 *daf-3*(*mgDf90*) *X*; *pdrEx42* [*osm-9(mutation 2)::gfp*;

*unc-122p::dsRed*]

SH227 *daf-5*(*e1386*) *II*; *pdrEx41* [*osm-9(mutation 2)::gfp*;

*unc-122p::dsRed*]

SH228 *daf-5*(*e1386*) *II*; *pdrEx42* [*osm-9(mutation 2)::gfp*;

*unc-122p::dsRed*]

SH230 *osm-9*(*ky10*) *IV*; *pdrEx29* [*sre-1*p::*osm-9 cDNA*::*gfp*;

*unc-122*p::*dsRed*]

SH231 *osm-9*(*ky10*) *IV*; *pdrEx30* [*sre-1*p::*osm-9 cDNA*::*gfp*;

*unc-122*p::*dsRed*]

SH233 *pdrEx31* [*sre-1*p::*osm-9 cDNA*::*gfp*; *unc-122*p::*dsRed*]

SH234 *pdrEx32* [*sre-1*p::*osm-9 cDNA*::*gfp*; *unc-122*p::*dsRed*]

SH237 *pdrEx34* [*osm-9p(deletion 1)::gfp; unc-122p::dsRed*]

SH239 *otIs24* [*sre-1p::gfp*]

SH240 *pdrEx35* [*osm-9p(deletion 1)::gfp; unc-122p::dsRed*]

SH241 *pdrEx36* [*osm-9p(deletion 2)::gfp; unc-122p::dsRed*]

SH242 *pdrEx37* [*osm-9p(deletion 3)::gfp; unc-122p::dsRed*]

SH243 *pdrEx38* [*osm-9p(deletion 3)::gfp; unc-122p::dsRed*]

SH244 *pdrEx39* [*osm-9p(mutation 1)::gfp; unc-122p::dsRed*]

SH245 *pdrEx40* [*osm-9p(mutation 1)::gfp; unc-122p::dsRed*]

SH246 *pdrEx41* [*osm-9p(mutation 2)::gfp; unc-122p::dsRed*]

SH247 *pdrEx42* [*osm-9p(mutation 2)::gfp; unc-122p::dsRed*]

SH248 *pdrEx43* [*osm-9p(mutation 3)::gfp; unc-122p::dsRed*]

SH249 *pdrEx44* [*osm-9p(mutation 3)::gfp; unc-122p::dsRed*]

SH250 *pdrEx45* [*osm-9p(mutation 4)::gfp; unc-122p::dsRed*]

SH251 *pdrEx46* [*osm-9p(mutation 4)::gfp; unc-122p::dsRed*]

SH252 *pdrEx47* [*osm-9p(mutation 5)::gfp; unc-122p::dsRed*]

SH253 *pdrEx48* [*osm-9p(mutation 6)::gfp; unc-122p::dsRed*]

SH254 *pdrEx49* [*osm-9p(mutation 6)::gfp; unc-122p::dsRed*]

SH255 *pdrEx50* [*osm-9p(mutation 7)::gfp; unc-122p::dsRed*]

SH256 *pdrEx51* [*osm-9p(mutation 7)::gfp; unc-122p::dsRed*]

SH257 *daf-5*(*e1386*) *II*; *daf-3*(*mgDf90*) *X*; *pdrEx41* [*osm-9(mutation 2)::gfp*;

*unc-122p::dsRed*]

SH258 *daf-5*(*e1386*) *II*; *daf-3*(*mgDf90*) *X*; *pdrEx42* [*osm-9(mutation 2)::gfp*;

*unc-122p::dsRed*]

SH265 *mut-16(mg461) I; otIs24 [sre-1p:;gfp]*

VC983 *hda-2(ok1479)* *II*

WM158 *ergo-1(tm1860)* *V*

WM193 *csr-1(tm892)* IV; *neIs20* [*pie-1::3xFLAG::csr-1 + unc-119(+)*]

WM206 *drh-3(ne4253)* *I*

WM27 *rde-1(ne219)* *V*

WM29 *mut-8/rde-2(ne221)* *I*

WM30 *mut-2/rde-3(ne298)* *I*

WM49 *rde-4(ne301)* *III*

WM53 *alg-2(ok304)* *II*

YY158 *nrde-3(gg66)* *X*

YY160 *nrde-1(gg88)* *III*

YY453 *nrde-4(gg129)* *IV*

VC2457 *ctn-1(gk3037)* *eri-6&C41D11.6(gk3038) I*; *gcy-20(gk1227) V*
